# Adsorptive Removal of Phosphate from Aqueous Solutions Using Low-Cost Volcanic Rocks: Kinetics and Equilibrium Approaches

**DOI:** 10.3390/ma14051312

**Published:** 2021-03-09

**Authors:** Dereje Tadesse Mekonnen, Esayas Alemayehu, Bernd Lennartz

**Affiliations:** 1 School of Chemical Engineering, Jimma Institute of Technology, Jimma University, Jimma P.O. Box 378, Ethiopia; getdere@gmail.com; 2 Faculty of Agricultural and Environmental Sciences, University of Rostock, Justus Von-Liebig Weg 6, 18059 Rostock, Germany; 3 Faculty of Civil and Environmental Engineering, Jimma Institute of Technology, Jimma University, Jimma P.O. Box 378, Ethiopia; 4 Africa Center of Excellence for Water Management, Addis Ababa University, Addis Ababa P.O. Box 1176, Ethiopia

**Keywords:** adsorption kinetics, aqueous solution, eutrophication, isotherm models, pumice, scoria

## Abstract

The contamination of surface and groundwater with phosphate originating from industrial and household wastewater remains a serious environmental issue in low-income countries. Herein, phosphate removal from aqueous solutions was studied using low-cost volcanic rocks such as pumice (VPum) and scoria (VSco), obtained from the Ethiopian Great Rift Valley. Batch adsorption experiments were conducted using phosphate solutions with concentrations of 0.5 to 25 mg·L^−1^ to examine the adsorption kinetic as well as equilibrium conditions. The experimental adsorption data were tested by employing various equilibrium adsorption models, and the Freundlich and Dubinin-Radushkevich (D-R) isotherms best depicted the observations. The maximum phosphate adsorption capacities of VPum and VSco were calculated and found to be 294 mg·kg^−1^ and 169 mg·kg^−1^, respectively. A pseudo-second-order kinetic model best described the experimental data with a coefficient of correlation of R^2^ > 0.99 for both VPum and VSco; however, VPum showed a slightly better selectivity for phosphate removal than VSco. The presence of competitive anions markedly reduced the removal efficiency of phosphate from the aqueous solution. The adsorptive removal of phosphate was affected by competitive anions in the order: HCO_3_^−^ >F^−^ > SO_4_^−2^ > NO_3_^−^ > Cl^−^ for VPum and HCO_3_^−^ > F^−^ > Cl^−^ > SO_4_^−2^ > NO_3_^−^ for VSco. The results indicate that the readily available volcanic rocks have a good adsorptive capacity for phosphate and shall be considered in future studies as test materials for phosphate removal from water in technical-scale experiments.

## 1. Introduction

Phosphate, a molecule consisting of the elements phosphorus (P) and oxygen [1], is an essential macronutrient for all life [2]. With nitrogen and carbon, phosphorus is the primary source of productivity and is fundamental for freshwater ecosystems’ sustainability [3]. However, a high phosphate concentration above 0.1 mg·L^−1^ [4] in water bodies has serious environmental repercussions, such as eutrophication, which is associated with massive algal growth, the excessive growth of microorganisms, and the depletion of dissolved oxygen (DO) in the water bodies, and, in turn, it harmfully effects life in the aquatic ecosystems [5,6,7,8,9]. However, some researchers reported that a phosphate concentration as low as 0.05 mg·L^−1^ [10] or 0.02 mg·L^−1^ [11] in the water reservoir is sufficient to stimulate the growth of algae. Nowadays, the contamination of water bodies by pollutants like phosphorus has drawn attention globally. Industrialization, modern agriculture, and human activities are the primary sources that can pose a serious threat to freshwater ecosystems due to the pollutants released from these into the environment [12].

In many cases, pollutants from different point and non-point sources infiltrate at high concentrations into the nearby water bodies [13]. Of the pollutants coming into water bodies, phosphate laden-water is the primary trouble of developing countries such as Ethiopia due to the formation of eutrophication in the surface water. The phosphate effluents from institutions, domestic activities, municipal activities, detergent-making industries, and agricultural-related activities are also the leading causes of eutrophication [14]. Wastewater released from slaughtering houses, for example, contains about 25–200 mg·L^−1^ phosphate [11].

The elimination of phosphate from water can be achieved via many processes, such as biological process, chemical precipitation, and adsorption methods [12,15]. In comparison, biological processes and chemical precipitation are expensive and result in a large amount of waste sludge to dispose of, involving high operating costs [16,17]. Therefore, it is of great interest to develop reliable technologies that utilize viable adsorbents to remove excess phosphate from water. It has been reported that due to its ease of application, economic feasibility, and high efficiency among the mentioned methods, adsorption is the most efficient process for the removal of phosphate from water [2,18].

Currently, a series of different adsorbents has been investigated for phosphate removal from synthesized aqueous water with or without surface modification. Some of these include zirconium oxide nanoparticles [19], exfoliated vermiculites [20], lanthanum hydroxide materials [21], bio-chars derived from crop straws [22], rice husk ash [23]; building waste [24], treated natural clinoptilolite [25], modified wheat straw [26], quaternary ammonium Chinese reed [27], zirconia-coated magnetite nanoparticles [28], corn stalks [29], iron oxide waste [30], and magnetic coal [31]. However, many of these suffer from either poor regenerablity or high process costs or low adsorption capacity. In such cases, looking for the most low-cost, easily available, and easily accessible raw materials that can be used as adsorbents is a critical concern for developing countries such as Ethiopia.

Volcanic rocks are inorganic adsorbents that are available all over the world, as well as in Africa. Pumice (VPum) and scoria (VSco) are abundant volcanic rock materials found in many parts of the world [32]. A vast amount of volcanic rocks are found in Ethiopia’s Great Rift Valley, which covers about 30% of the area of the country [33,34]. Due to their vascular structure, high contents of silica, alumina, iron oxides and alkaline earth metals, large surface areas, and environmental friendliness, VPum and VSco are given conspicuous roles for pollutants removal (i.e., phosphate ions) [16,35]. Previous studies have showed the adsorption efficiency of volcanic rocks (pumice and scoria) with heavy metals [36,37,38,39], fluoride [40], and phosphorus [41] in a single system. In Ethiopia, volcanic rocks are also applied to remove hazardous pollutants from tannery wastewater [39], but there is little information on which raw volcanic rocks have been applied to remove phosphate from aqueous solutions. As such, the objectives of this study were (i) to investigate the recovery of phosphates from aqueous solution using volcanic rocks via classical slurry batch experiments, (ii) to determine the adsorption kinetics and adsorption isotherm models to compare the removal efficiency of the adsorbents (VPum and VSco), and (iii) to evaluate the impact of competitive/co-existing anions on the removal of phosphate.

## 2. Materials and Methods

### 2.1. Materials Preparation and Characterization

The volcanic rock materials, VPum and VSco, were obtained from the Great Rift Valley of Ethiopia. Before the experiments, the rock materials were washed several times using deionized water to remove dust, dirt, and adhering particles from the adsorbent’s surface, and then dried for 24 h at 105 °C in the oven. The well-dried materials were ground to a powder form and sieved to the size of 0.075–0.425 mm. The well dried and ground materials were then coated with iron salts using iron (II) chloride tetrahydrate (FeCl_2_·4H_2_O) to promote cations on the adsorbents’ surface. Forty grams of the adsorbents were soaked with enough FeCl_2_·4H_2_O solution at a concentration of 0.01 M. The mixture was then sonicated at room temperature for 7 h using an ultrasonic mixer (BANDELIN SONOREX RK 16, Retsch GmbH, Haan, Germany). After sonication mixing, the decanted rock materials were heated at 90 °C for 18 h, then cooled and washed with deionized water several times until the suspension became clean, and then dried again at 105 °C in the oven for 24 h. The well-prepared adsorbent materials were labeled and stored in a desiccator until the next experiments resumed.

Proximate and ultimate analyses of the adsorbent materials (VPum and VSco) were performed according to our previous research [42]. The pH zero point charge (pHzpc) of adsorbent materials was measured according to [43] using a solid addition method. The materials’ organic matter and moisture content were measured according to our previous study as well [42], wherein the particle density and specific surface area (BET) of the rock materials were measured according to another previous study [44]. The organic matter, moisture content and pHzpc of the VPum were 4.95%, 0.85%, and 9.2, and those of the VSco were 0.21%, 0.45%, and 7.2, respectively. The specific surface area and particle density of the VPum were 3.5 m^2^·g^−1^ and 2.46 g·cm^−3^, and those of the VSco were 2.48 m^2^·g^−1^ and 2.98 g·cm^−3^, respectively. A scanning electron microscope equipped with an energy dispersive X-ray (SEM-EDX) was employed to analyze the adsorbents’ surface morphology and the microstructure of the adsorbent materials. All the chemicals and reagents used in this study were of analytical grade unless otherwise specified. Table 1 shows that the chemical composition of raw VPum and VSco, measured by X-ray fluorescence (XRF) spectrometry, which was performed according to [33]. The greater the presence metal ions such as Si and Fe, the more easily the materials attract phosphate ions by forming surface charges.

### 2.2. Batch Adsorption Studies

The equilibrium batch experiments were carried out to determine the removal capacities and the percentage removal of phosphate with the VPum and VSco. The procedures described in our previous work [42] were followed to prepare a working solution ranging from 0.5 to 25 mg·L^−1^, representing the minimum to typical phosphate concentration in water, by diluting a stock solution of potassium dihydrogen phosphate (KH_2_PO_4_). An initially known amount of each adsorbent was added to 10 mg·L^−1^ phosphate (1:5 solid to solution ratio) in 100 mL plastic flasks. The initial pH of each solution throughout the experiments was adjusted to 6 ± 0.5 and 5.0 ± 0.5 for VPum and VSco, respectively, at which levels monoanionic phosphate species exists. According to the repartition diagram of phosphate species, at the studied pH ranges (pH = 6.5 for VPum and pH = 5.5 for VSco), the phosphate species that predominantly exists could be H_2_PO_4_^−^ [45].

The well-capped plastic flasks containing the mixtures of adsorbents and adsorbate were then placed on a horizontal mechanical shaker operating at 200 rpm and at room temperature. After 420 min of mixing, the mixtures were filtered through a 0.45 µm filter, and the concentration of phosphate in the filtrate was analyzed using a continuous flow analyzer (CFA) (AA3 from seal Analytical, GmbH, Norderstedt, Germany). Subsequently, the amount of adsorbed phosphate at equilibrium (q_e_) and the percentage removal (A%) of phosphate from the aqueous solution were calculated using Equations (1) and (2), respectively:

(1) $$q_{e}=\frac{\left(C_{o}-C_{e}\right)V}{M}\;$$

(2) $$\left(A\%\right)=\left(1-\frac{C_{e}}{C_{o}}\right)\times 100\;$$

where q_e_ is the amount of phosphate adsorbed (removal capacity) per unit mass of adsorbent at equilibrium; Co and Ce are the initial and final concentrations of phosphate at equilibrium (mg·L^−1^), respectively; V is the volume of the solution contacted with the adsorbent (L); M is mass of the adsorbent (g) and (A%) is percentage removal of phosphate at time t.

Similarly, phosphate’s adsorption kinetics on the samples were evaluated by mixing 8 g of the VPum and VSco samples with 200 mL of solution at 10 mg·L^−1^ phosphate concentration in 250 mL plastic flasks. The mixtures were then shaken on the horizontal mechanical shaker, then the samples were withdrawn at each predetermined interval of time (5, 10, 20, 30, 40, 60, 90, 120, 180, 240, 300, and 420 min). At the end of each experiment, the mixture was immediately filtered using filter paper, and then phosphate analysis was carried out using CFA.

The effects of co-existing anions (nitrate (NO_3_^−^), sulfate (SO_4_^2−^), bicarbonate (HCO_3_^−^), chloride (Cl^−^) and fluoride (F^−^) from their respective salts of KNO_3_, Na_2_SO_4_, NaHCO_3_, NaCl and NaF, respectively) on the removal of phosphate were determined in a separate experiment. First, solutions were prepared from the respective salts at 0.1 M concentration using deionized water in a separate bottle. Then, 5 mL equal concentrations (0.1 M) of each anion were mixed with 20 mL of phosphate solution at 10 mg·L^−1^ concentration in a series of 100 mL Erlenmeyer plastic flasks. Then, 1 g of the adsorbents was added to a series of plastic flasks. The flasks were well capped and shaken on the horizontal shaker working at 200 rpm for 24 h at room temperature. The final suspension solution was collected and filtered, and then the phosphate concentration was measured with similar methods.

### 2.3. Adsorption Equilibrium Studies

To understand the quantity of adsorbates that are accommodated by the adsorbents’ surface, adsorption equilibrium information is critical. The equilibrium of adsorption between solution and adsorbent is well described by different non-linear and linear forms of adsorption isotherms [46]. Langmuir, Freundlich, and Dubinin–Radushkevich (D-R) isotherms were employed to describe the equilibrium between the adsorbate and adsorbent for experimental data. The Langmuir adsorption isotherm describes the formation of monolayer adsorption of the adsorbate on the surface of the adsorbent [47], and this can be expressed by Equations (3) and (4):

(3) $${\;q}_{e}=\frac{q_{m}K_{L}C_{e}}{1+K_{L}C_{e}}\;\left(\mathrm{non}-\mathrm{linear}\;\mathrm{form}\right)$$

(4) $$\frac{C_{e}}{q_{e}}=\frac{1}{q_{m}K_{L}}+\frac{C_{e}}{q_{m}}\;\left(\mathrm{linear}\;\mathrm{form}\right)$$

where q_e_ (mg·g^−1^) is equilibrium adsorption capacity; C_e_ (mg·L^−1^) is the equilibrium concentration of the adsorbate in the solution; q_m_ (mass of adsorbate per mass of adsorbent) is the maximum adsorption capacity of phosphate; K_L_ (L·mg^−1^) is the Langmuir isotherm constant. The constants K_L_ and q_m_ can be calculated from the intercept and slope of the linear plot of C_e_/q_e_ vs. C_e_ (Figure S1 in the supplementary data). The essential characteristics, feasibilities, and shapes of the Langmuir isotherm can be described by a dimensionless constant, the so-called separation factor, R_L_ (Equation (5)) [48]:

(5) $$R_{L}=\;\frac{1}{1+K_{L}C_{o}}$$

where K_L_ and Co are Langmuir constant (L·mg^−1^) and initial phosphate concentration (mg·L^−1^) in the solution. The shape of the isotherm is determined by the values of the R_L_; as R_L_ = 0 indicates irreversible adsorption, and R_L_ = 1 shows linear adsorption, where R_L_ > 1 and 0 < RL < 1 indicate unfavorable and favorable adsorption, respectively, as indicated by [33,48].

The Freundlich isotherm involves heterogeneous adsorption or multi-layer adsorption [49], and can be described by Equations (6) and (7):

(6) $$q_{e}={\;K}_{F}C_{e}{}^{1/n}\;\left(\mathrm{non}-\;\mathrm{linear}\;\mathrm{form}\right)\;$$

(7) $${\mathrm{lnq}}_{e}={\mathrm{lnK}}_{F}+\frac{1}{n}{\mathrm{lnC}}_{e}\;\left(\mathrm{linear}\;\mathrm{form}\right)$$

where q_e_ (mg·g^−1^) is the equilibrium adsorption capacity; C_e_ (mg·L^−1^) is the equilibrium concentration of the adsorbate in the solution; K_F_, and n (unit-less) are the Freundlich isotherm constants related to sorption capacity and sorption energy, respectively. Freundlich isotherm’s constants can be calculated from the slope and intercept of the linear plot of lnq_e_ vs. lnC_e_ (Figure S2 in supplementary materials). The values of “n” indicate the type of adsorption isotherm; when the value of 1/n is greater than zero, which means 0 < 1/n < 1, the adsorption is favorable and chemisorption occurs. When 1/n is greater than one, the adsorption is unfavorable, and irreversible adsorption occurs if 1/n is equal to unity [50].

The Dubinin-Radushkevich (D-R) isotherm is an empirical model that does not assume homogenous or constant sorption potential. It instead estimates the solute’s mean free energy change when a mole of solute in the solution is transferred to the surface of the adsorbent [43,51]. The mean free energy describes the physical or chemical nature of the reaction for the adsorption. The D-R isotherm can therefore be expressed by Equation (8):

(8) $${\mathrm{lnq}}_{e}={\mathrm{lnq}}_{\max\;}-{\;\beta \varepsilon }^{2}\;$$

where q_e_ is the equilibrium adsorption capacity (mol·g^−1^), q_max_ is the maximum adsorption capacity (mol·g^−1^), β is the activity coefficient or adsorption constant depending on the mean free energy of adsorption, and ε is the Polanyi adsorption potential, which can be expressed by Equation (9):

(9) $$\varepsilon =\mathrm{RTln}\left(1+\frac{1}{C_{e}}\right)\;$$

where R is the universal gas constant (kJ·mol^−1^·K^−1^), T is the absolute temperature (K), and C_e_ is the equilibrium concentration of the adsorbate in the solution (mol·L^−1^). The q_max_ and β can be obtained from the linear plot of lnq_e_ vs. $\varepsilon^{2}$ (Figure S3). The mean free energy of adsorption E (kJ·mol^−1^) can be obtained from the expression of Equation (10) [52]:

(10) $$E=\frac{1}{\sqrt{\left(2\beta \right)}}$$

### 2.4. Adsorption Kinetics

To quantify the time-dependent adsorption process, adsorption kinetics were investigated using pseudo-first-order kinetics, pseudo-second-order kinetics, and intra-particle diffusion models.

Lagergren pseudo-first-order expression is given by Equation (11) [43]:

(11) $$\ln(q_{e}-{\;q}_{t})=\ln\left(q_{e}\right)-K_{1}t$$

where K_1_ is the first-order rate constant of adsorption (min^−1^); q_e_ (mg·g^−1^) and q_t_ (mg·g^−1^) are the amount of phosphate adsorbed at equilibrium and at time t, respectively. The values of K_1_ and q_e_ can be obtained from the slope and the intercept of a linear straight-line plot of ln(q_e−_q_t_) vs. t.

The pseudo-second-order expression can be described by Equations (12) and (13):

(12) $$\frac{{\mathrm{dq}}_{t}}{\mathrm{dt}}=K_{2}{\left(q_{e}-q_{t}\right)}^{2}$$

which can be integrated via the linear expression as:

(13) $$\frac{t}{q_{t}}=\frac{1}{h}+\;\frac{1}{q_{e}}t\;$$

where q_e_ (mg·g^−1^) and q_t_ (mg·g^−1^) are the amount of phosphate adsorbed at equilibrium and time t, respectively; h = K_2_qe^2^ and K_2_ is the rate constant of the pseudo-second-order model (g·mg^−1^·min^−1^). The values of K_2_ and q_e_ can be obtained, respectively, from the intercept and slope of the linear plot t/q_t_ vs. t.

The intra-particle diffusion equation can be expressed as shown in Equation (14) [2]:

(14) $$q_{t}=\;K_{id}t^{0.5}+C\;$$

where q_t_ is the amount of phosphate adsorbed at time t, K_id_ (mg·g^−1^·min^−1/2^) is the intra-particle diffusion rate constant obtained from the slope of the plot q_t_ vs. t^0.5^, and C (mg·g^−1^) is the intercept of the plot, often referred to as the thickness of the boundary layer and obtained from the intercept of the plot [53].

## 3. Results and Discussions

### 3.1. Adsorbents Characterization

**Figure 1:**
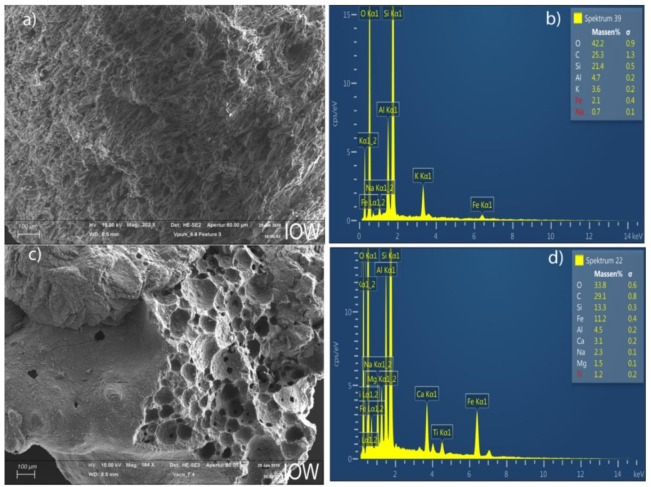
Scanning electron microscope (SEM) and energy dispersive X-ray (EDX) patterns of Vpum (**a**,**b**) and Vsco (**c**,**d**), respectively.

The scanning electron microscope (SEM) analysis revealed that the surface morphology of the VPum was rough and wrinkled, which potentially provides more adsorption sites for phosphates (Figure 1a) than VSco, where a relatively smooth and uniform surface structure was observed (Figure 1c). The pumice’s porousness and cavities indicated that the pumice materials had a rougher surface compared to scoria, which implies that pumice has a better phosphate adsorption capacity than scoria. Figure 1b,d show the energy dispersive X-ray (EDX) image for the adsorbent’s elemental composition of VPum and VSco. The measured EDX image indicates that the oxides of Al, Si, Fe, and K were the main constituents of the adsorbent materials, while the rest of the elements, such as Mn and Pb, were detected at lower values (<0.1% weight).

### 3.2. Effects of Contact Time

**Figure 2:**
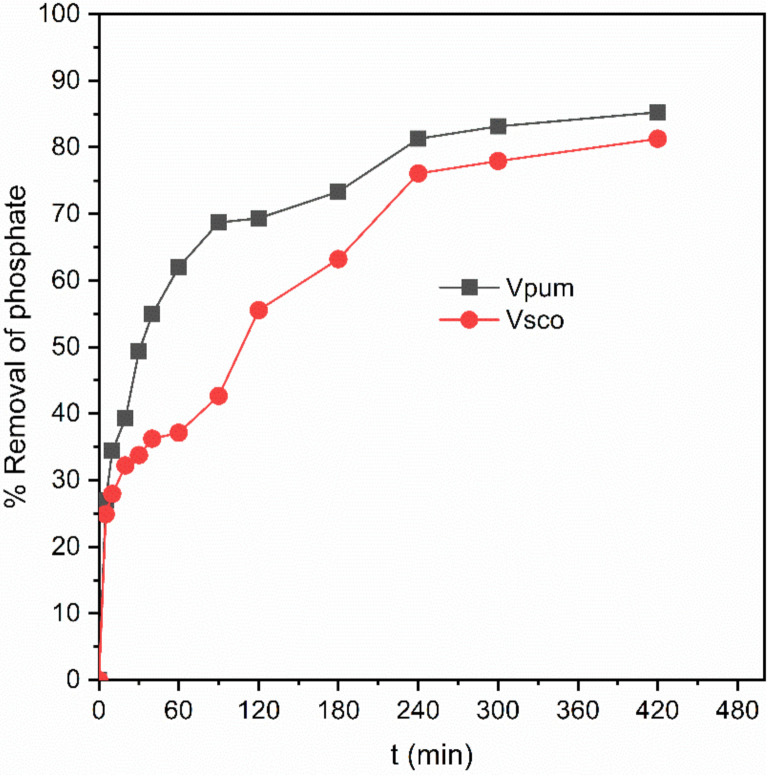
Effect of contact time on the removal efficiency of phosphate.

Designing an appropriate batch adsorption experiment is very important to get the rate at which the adsorption takes place, and the adsorption process’ time dependence was described by varying the contact time between the adsorbates and the adsorbents’ surface. Twelve contact points were examined to evaluate the effects of contact time on the abilities of VPum and VSco to remove phosphate at room temperature. The impact of contact time on the percent of removed phosphate is shown in Figure 2. The phosphate removal efficiencies of VPum and VSco were sharply increased for the first 240 min, from 0 to 81% for VPum and from 0 to 76% for VSco. The first fast increase in the removal efficiency was due to the significant validity of active free pore sites on the adsorbent’s surface compared to the remaining adsorption time [54,55]. As time goes on, the adsorbent’s surface gets saturated with phosphate, and only a few phosphate removals occurred during the last 3 h of equilibrium. Therefore, equilibrium was achieved at the 420 min contact time. Similar materials were used to remove chromium from the aqueous solution, and adsorption equilibrium was attained at 540 min [38]. However, a fast equilibrium time was attained at 120 min for blended rock materials with chitosan, used to remove arsenic from aqueous solution [32]. This indicates that the surface modification of the adsorbents provides a better adsorption time, as the contact time is crucial for calculating the process’ optimum reaction time [29].

It was also observed that the removal efficiency of VPum was slightly better than that of VSco, which was in agreement with the results for the adsorbents’ surface morphology obtained from SEM. The more porous the surface is, the better the phosphate’s adsorption onto the adsorbents’ surfaces [56].

### 3.3. Adsorption Kinetics

**Figure 3:**
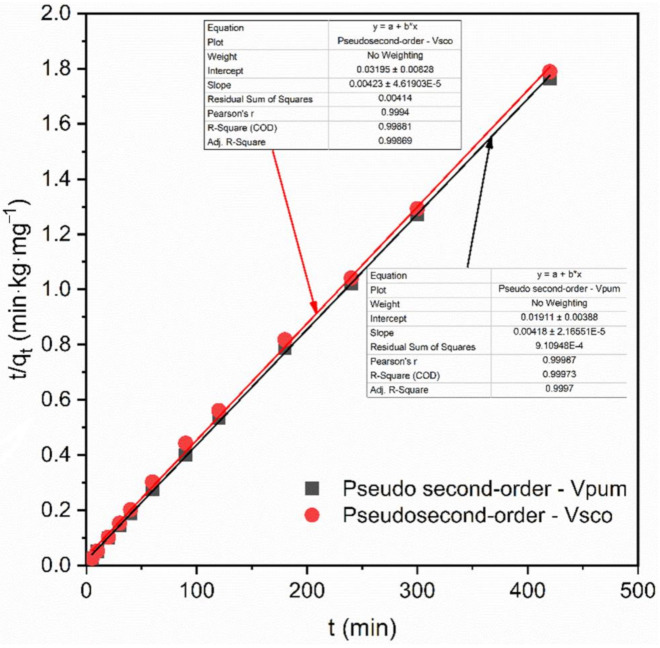
Pseudo-second-order kinetic plot for VPum and VSco.

**Figure 4:**
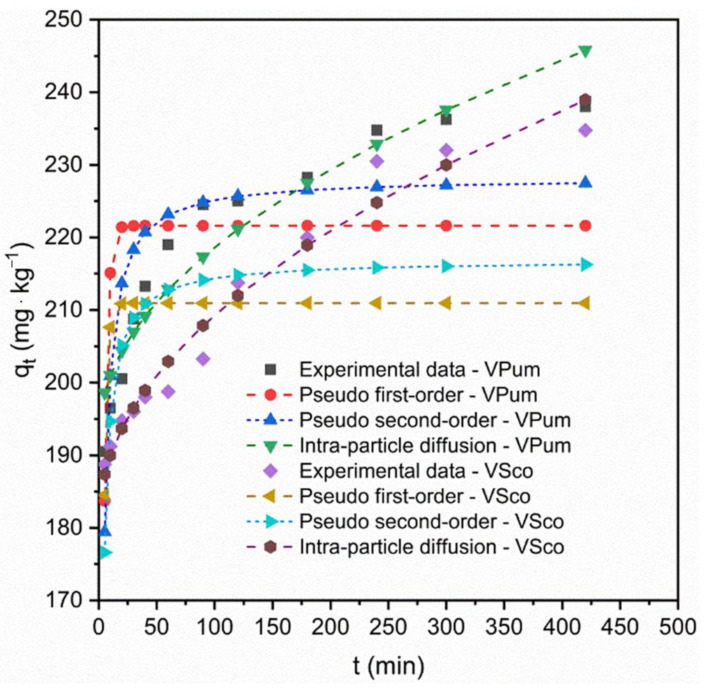
Kinetics of phosphate adsorption on VPum and VSco, and data fitting for pseudo-first-order, pseudo-second-order, and intra-particle diffusion.

Due to providing worthy information on the reaction pathways and adsorption mechanisms, adsorption kinetics analysis plays a vital role in the adsorption system [57]. The mass transfer and chemical reaction mechanisms of the adsorptive removal of phosphate onto VPum and VSco can be examined by various kinetic models, namely Lagergren pseudo-first-order, pseudo-second-order, and Weber and Morris intra-particle diffusion kinetic models [58,59]. Therefore, their validity for use with the experimental adsorption data for phosphate onto VPum and VSco was investigated. The results obtained from the pseudo-second-order model exhibited higher correlation values (R^2^ > 0.99) for both VPum and VSco than the pseudo-first-order model tested in this study. Moreover, the calculated adsorptive capacity values (q_e, cal_) from the pseudo-second-order kinetic model were 228.22 mg·kg^−1^ and 216.84 mg·kg^−1^ for VPum and VSco, respectively. These values agreed well with the experimentally determined adsorptive capacity values (q_exp_) of 238 mg·kg^−1^ and 234.75mg·kg^−1^ for VPum and VSco, respectively. Moreover, the pseudo-second-order linear plot (Figure 3) shows the good agreement of the experimental data with the calculated data for VPum and VSco. Nevertheless, the values of the calculated removal capacity and its linear plot (Figure S4) obtained from pseudo-first-order, compared to pseudo-second-order, for both VPum and VSco were not allied with the values of the removal capacity derived from the experimental values. This validates that the pseudo-first-order kinetic model does not adequately describe the adsorption process, whereas the adsorptive removal of phosphate by VPum and VSco followed the pseudo-second-order reaction [60,61]. The non-linear adsorption kinetics plots for pseudo-first-order, pseudo-second-order, and intra-particle diffusion are shown in Figure 4, which describes the nonlinear curve fitting of the adsorption kinetics and its corresponding constant parameters, and the calculated and experimental values of the adsorption kinetic models are summarized in Table 2.

**Figure 5:**
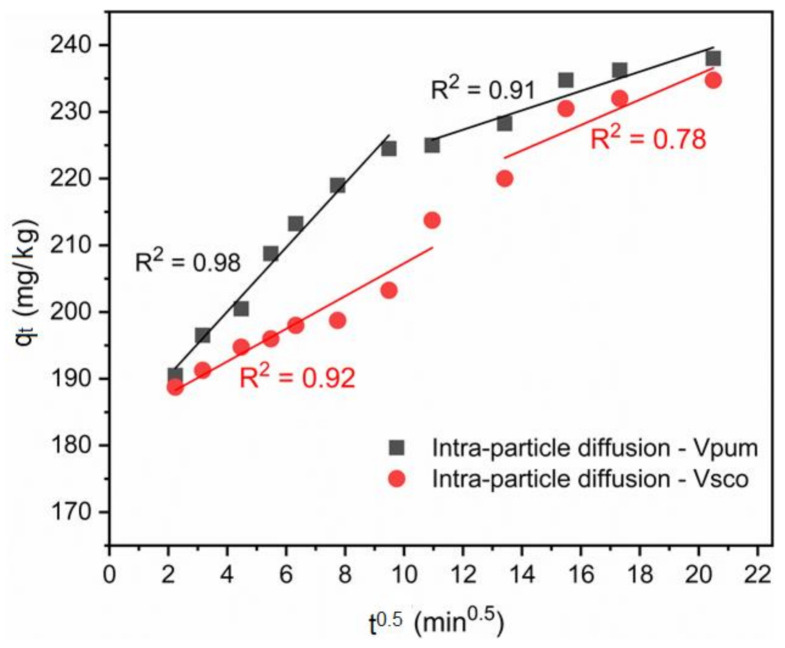
Intra-particle diffusion plot for VPum and VSco with two different slopes.

Intra-particle diffusion is also responsive to the diffusion mechanism of the adsorbate in the solution. If the plots for the intra-particle diffusion q_t_ vs. t^0.5^ of Equation (14) both for VPum and VSco are leaner and pass through the origin, then the intra-particle diffusion model is the sole rate-limiting step; otherwise, an adsorptive process other than intra-particle diffusion can occur [61]. According to the intra-particle diffusion model, three points can be valid: (i) if the plot of q_t_ vs. t^0.5^ is linear, the intra-particle diffusion model will be involved in the rate-controlling step for the adsorption process; (ii) if this line passes through the origin, i.e., intercept = 0, then the intra-particle diffusion model is the sole rate-controlling step of the adsorption process, and (iii) if two or more slopes could occur, then there should be an indicator for the multi-step adsorption process [62]. In our case, the linear plot of q_t_ vs. t^0.5^ (Figure 5) did not pass through the origin, but was linear in some points, and two slopes were observed. Therefore, this validates that intra-particle diffusion was not the only rate-limiting step, and non-diffusive reaction also occurred between the phosphate ion and rock materials. This applicability of the intra-particle diffusion model for the present kinetic data is also in agreement with other studies [62,63].

### 3.4. Effects of Initial Concentration

The variation in phosphate removal by VPum and VSco as a function of the initial phosphate concentration is presented in Figure S5 in the supplementary material. It was noticed that the percent removal of phosphate decreased (from 93% to 43% and from 81% to 23%, for VPum and VSco, respectively) with an increase in initial phosphate concentration (from 0.5 to 25 mg·L^−1^). This is because the resistance to the up-taking of the adsorbate onto the adsorbents’ surfaces is higher, and the active site for adsorption gets saturated at higher concentrations [10]. In contrast, phosphate removal capacity, q_t_, increased with increasing the initial phosphate concentration due to the higher driving forces provided by the higher initial concentrations, which were able to resist the mass transfer between the solid surface and aqueous solution [59], which enables the adsorbate to remain on the surface of the adsorbents. Furthermore, for the initial phosphate concentration in the range of 5 to 25 mg·L^−1^, the amount of phosphate adsorbed decreased in the order of VPum > VSco. This revealed that VPum has, relatively, a better removal capacity than VSco.

### 3.5. Adsorption Isotherm

**Figure 6:**
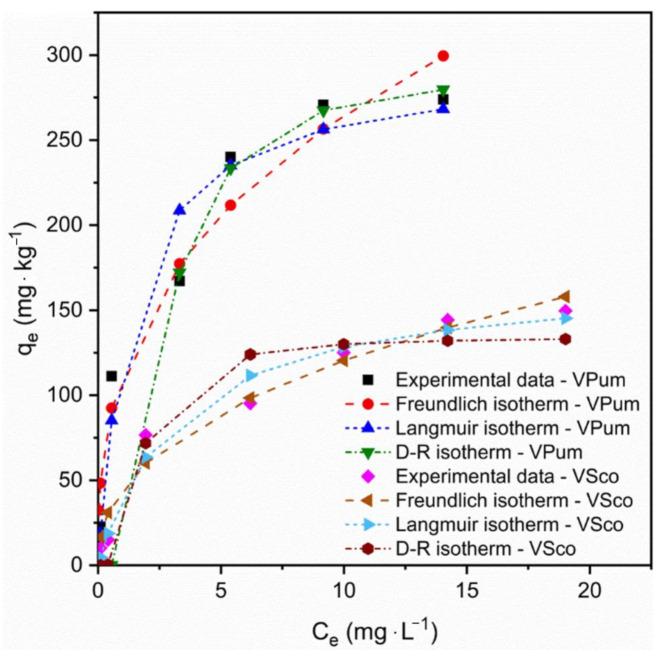
Nonlinear phosphate adsorption isotherms for VPum and VSco.

The adsorption equilibrium data are represented with the correspondences of adsorption capacity, qe, and the adsorbate’s concentration, Ce, at equilibrium. Figure 6 shows the isotherm plots and experimental data. As seen in Figure 6, the adsorption capacity qe increases with the increase in phosphate concentration. This is due to the increased mass transfer driving force with the increasing initial phosphate concentrations [29]. The constant values and coefficients of determination of the adsorption isotherms are described in Table 3. From the results obtained in Table 3, R_L_ values for the Langmuir and 1/n values for the Freundlich isotherms were less than unity, and this elucidates that the adsorption of phosphate onto VPum and VSco is favorable. Furthermore, the lower value of 1/n (0 < 1/n < 1) is also a good indicator that chemisorption was governing the adsorption process for VPum and VSco. Similar findings were reported in the literature [50,51].

The adsorption behavior can be depicted as physical or chemical, depending on the value of the mean free energy (E) of the D-R isotherm model. When the mean free energy of adsorption is between 1 and 8 kJ·mol^−1^, the adsorption behavior can be depicted as physical adsorption. The force of attraction between the adsorbate and the adsorbent is feeble. Otherwise, it is represented as chemical adsorption if the value of the mean free energy of adsorption is greater than 8 kJ·mol^−1^. The chemical bond between the adsorbate and the adsorbent is very strong. From the results obtained in Table 3, we see that the value of E for the D-R isotherm is between 1 and 8 kJ·mol^−1^ for both VPum and VSco, indicating that the adsorption process was also governed by physical adsorption due to Van der Waal’s forces [48,59]. Unlike the Langmuir and Freundlich isotherm models, a D-R isotherm model follows the pore-filling mechanisms with the assumption of a multilayer character, which involves the Van der Waal’s force applied to the physical adsorption process. Still, from the adsorption kinetic values and Freundlich isotherm, we see that the adsorption process followed chemisorption. The adsorption process’ deviation confirmed phosphate adsorption onto VPum and VSco following the physisorption and chemisorption [50]. The covalent forces of electron attraction due to metal ions exist on the adsorbent’s surface, and surface attraction between the adsorbent and adsorbate is applied [64]. A similar study was reported wherein both physisorption and chemisorption were applied to remove orange 16 onto hemp stalk activated carbon [65].

### 3.6. Effects of Competitive Anions

**Figure 7:**
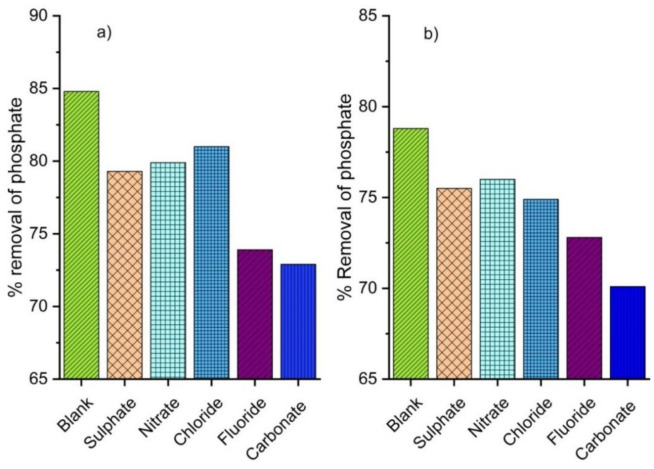
Effects of competitive anions on phosphate removal: (**a**) pumice and (**b**) scoria.

Real wastewater commonly contains different anions, which exist in various forms of concentration [66,67]. Phosphate adsorption onto VPum and VSco was independently studied in competing anions: nitrate (NO_3_), sulfate (SO_4_^2−^), chloride (Cl^−^), fluoride (F^−^) and bicarbonate (HCO_3_). The effects of competitive anions on phosphate removal onto the surface of the adsorbent materials showed that the phosphate removal efficiency of VPum and VSco with the absence of competitive anions was 85% and 79%, respectively (Figure 7). The reductions in phosphate removal efficiency were observed after adding the competitive anions. When 0.1 M of bicarbonate ion was introduced to the phosphate solution, the removal efficiency was reduced by 14.1% for VPum and 11.4% for VSco. The addition of the bicarbonate ion may make the overall solution of phosphate alkaline, by raising the pH (from 6.5 to 9.6 and from 5.5 to 7.8, for VPum and VSco, respectively), which could be the reason for the decrease in phosphate’s removal efficiency. In this case, an anion exchange reaction occurs between the phosphate ions and metal oxide, which favorably competes with hydroxide ions. The affinity of phosphate ions to the surface of the adsorbents becomes lower, and the adsorption efficiency is decreased. Similar findings were also reported in other studies [20,68]. Similarly, a fluoride ion has shown a high impact on the removal of phosphate onto VPum and VSco materials, whereby the removal efficiency of phosphate reduced by 13% and 7.6%, respectively. The noticeable reductions in phosphate removal efficiency by fluoride ion were likely due to the higher competition for fluoride ions binding to the adsorbents’ surface than for phosphate ions [69]. From this result, we can conclude that VPum and VSco as adsorbents can be applied for the simultaneous removal of phosphate and fluoride from water. However, no noticeable effect was observed due to chloride for VPum or nitrate for VSco materials. Similar studies reported that nitrate and chloride did not affect phosphate removal when bio-char from anaerobically digested sugar beet, silicate hybrid materials, and acid-coated magnetite nanoparticles was employed as the adsorbent, respectively [68,70,71]. In contrast, the addition of nitrate into the aqueous solution greatly affected the removal efficiency of phosphate onto the modified sludge bio-char adsorbent [72]. These contradictions in the present study’s results and previous studies might be attributable to different experimental conditions, such as anion concentration and other adsorbents’ properties.

## 4. Conclusions

In the present study, we used cost-effective, environmentally friendly, and easily accessible volcanic rock materials (pumice and scoria) to recover phosphate from aqueous solution. The Freundlich and Dubinin Radushkevich isotherms better described the adsorption process than the Langmuir isotherm model. The optimized parameter values for various adsorption kinetic models and adsorption isotherms advocate that the adsorption processes are mainly dominated by chemisorption and the strong attraction of covalent bonds. However, the value of mean free energy, as obtained from the Dubinin-Radushkevich isotherm, indicated that physisorption may likewise play an important role in the removal process due to Van der Waal forces. It was also observed that the removal of phosphate from the solution was dramatically affected by competitive anions. Thus, the simultaneous removal of pollutants has to be considered during water treatment. Overall, volcanic rocks (i.e., pumice and scoria) can be used as potential adsorbents to remove phosphate from water, but additional studies are required to further explore the potential of the rock material in a technical-scale (flow-through) set-up. In addition, several repetitions for the desorption and regeneration of the rock materials is highly recommended in further studies so as to increase the reusability of the materials.

## Figures and Tables

**Table 1:** Chemical compositions of pumice (VPum) and scoria (VSco) [33].

Chemical Compositions (wt.%)	SiO_2_	Al_2_O_3_	Fe_2_O_3_	CaO	K_2_O	Na_2_O	MgO	TiO_2_	Others
VPum	68.6	8.9	4.9	1.8	5.5	4.1	0.2	0.3	5.7
VSco	47.4	21.6	8.9	12.4	0.5	3.0	3.3	1.7	1.2

**Table 2:** Calculated and experimental values of parameters for pseudo-first order, pseudo-second order, and the intra-particle diffusion kinetics model for phosphate adsorption onto volcanic rocks.

Kinetic Model	Parameters	VPum	VSco
**Experimental**	q_exp_ (mg·kg^−1^)	238.0	234.75
**Pseudo-first-order**	q_e,cal_ (mg·kg^−1^)	221.60	210.94
	K_1_ (min^−1^)	0.35	0.42
	R^2^	0.97	0.98
**Pseudo-second-order**	q_e,cal_ (mg·kg^−1^)	228.22	216.84
	K_2_ (g·mg^−1^·min^−1^)	3.20	4.10
	R^2^	0.999	0.999
**Intra-particle diffusion**	K_1id_ (mg·kg^−1^·min^−1/2^)	4.82	2.45
	K_2id_ (mg·kg^−1^·min^−1/2^)	1.45	1.90
	C_1id_ (mg·kg^−1^)	180.81	182.78
	C_2id_ (mg·kg^−1^)	209.92	197.55
	R_1_^2^	0.98	0.92
	R_2_^2^	0.91	0.78

**Table 3:** Adsorption isotherm parameters for the adsorption of phosphate onto volcanic rocks.

Isotherms	Parameters	Adsorbents	
		Pumice	Scoria
Langmuir isotherm	K_L_ (L·mg^−1^)	0.74	0.31
	q_m_ (mg·kg^−1^)	294.28	169.95
	R^2^	0.73	0.81
	R_L_	0.05–0.7	0.11–0.87
Freundlich isotherm	K_F_ ((mg·kg^−1^)(mg·L^−1^)1/n)^−1^	114.80	45.58
	n	2.76	2.37
	R^2^	0.89	0.92
Dubinin-Radushkevich isotherm	q_max_ (mg·kg^−1^)	289.81	134.32
	E (kJ·mol^−1^)	3.31	5.44
	B	0.045	0.017
	R^2^	0.80	0.69

## Data Availability

The data used in this study can be accessed from the authors with reasonable requests.

## Supplementary Materials

The following are available online at https://www.mdpi.com/1996-1944/14/5/1312/s1, Figure S1: Linear plot of Langmuir isotherm for VPum and VSco, Figure S2: Linear plot of Freundlich isotherm model for VPum and VSco, Figure S3: Linear plot of Dubinin-Radushkevich isotherm model for VPum and VSco, Figure S4: Pseudo first-order kinetic plot for VPum and VSco, Figure S5: Effect of initial concentrations.
