# Supplementary material for: Adsorptive Removal of Phosphate from Aqueous Solutions Using Low-Cost Volcanic Rocks: Kinetics and Equilibrium Approaches
Source: Materials (Basel). 2021 Mar 9;14(5):1312. doi: 10.3390/ma14051312 (PMC7967176; doi:10.3390/ma14051312)
Supplement: Supplementary file 1 [file materials-14-01312-s001.pdf]

materials

MDPI

Supplementary Materials

Adsorptive Removal of Phosphate from Aqueous Solutions Using Low-Cost Volcanic Rocks: Kinetics and Equilibrium Approaches

Dereje Tadesse Mekonnen [1,2], Esayas Alemayehu [3,4,*] and Bernd Lennartz [2,*]

[1] Jimma Institute of Technology, Jimma University, School of Chemical Engineering, Jimma P.O. Box 378, Ethiopia; getdere@gmail.com
[2] Agricultural and Environmental Sciences, University of Rostock, Justus Von-Liebig Weg 6, 18059 Rostock, Germany
[3] Faculty of Civil and Environmental Engineering, Jimma Institute of Technology, Jimma University, Jimma P. O. Box 378, Ethiopia
[4] Africa Center of Excellence for Water Management, Addis Ababa University, Addis Ababa P.O. Box 1176, Ethiopia
* Correspondence: esayas16@yahoo.com (E.A.); bernd.lennartz@uni-rostock.de (B.L.); Tel.: +251-917-017-002 (E.A.); +49-381-498-3180 (B.L.)

Citation: Mekonnen, D.T.; Alemayehu, E.; Lennartz, B. Adsorptive Removal of Phosphate from Aqueous Solutions Using Low-Cost Volcanic Rocks: Kinetics and Equilibrium Approaches. *Materials* **2021**, *14*, 1312. https://doi.org/10.3390/ma14051312

Academic Editors: Ewa Skwarek and Agnieszka Gładysz-Płaska

Publisher's Note: MDPI stays neutral with regard to jurisdictional claims in published maps and institutional affiliations.

Copyright: © 2021 by the authors. Licensee MDPI, Basel, Switzerland. This article is an open access article distributed under the terms and conditions of the Creative Commons Attribution (CC BY) license (http://creativecommons.org/licenses/by/4.0/).

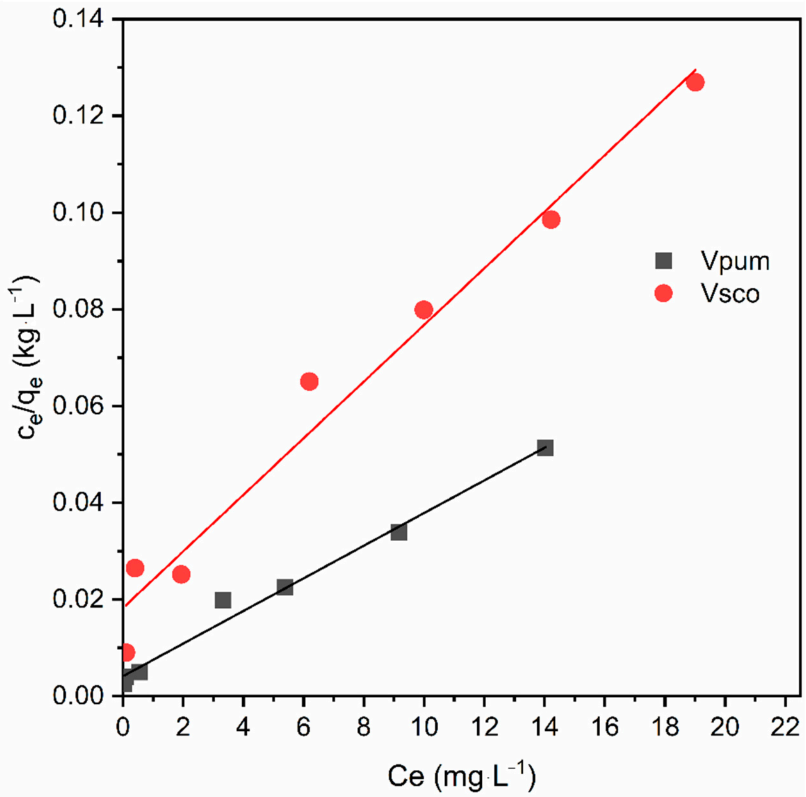

Figure S1. Linear plot of Langmuir isotherm for Vpum and Vsco.

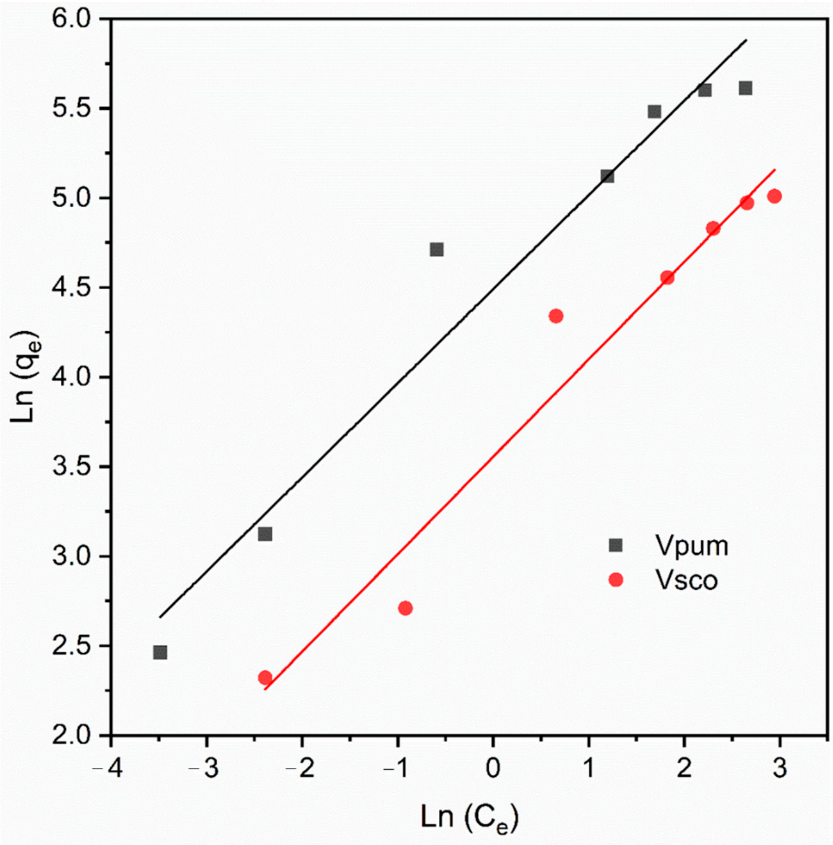

Figure S2. Linear plot of Freundlich isotherm model for Vpum and Vsco.

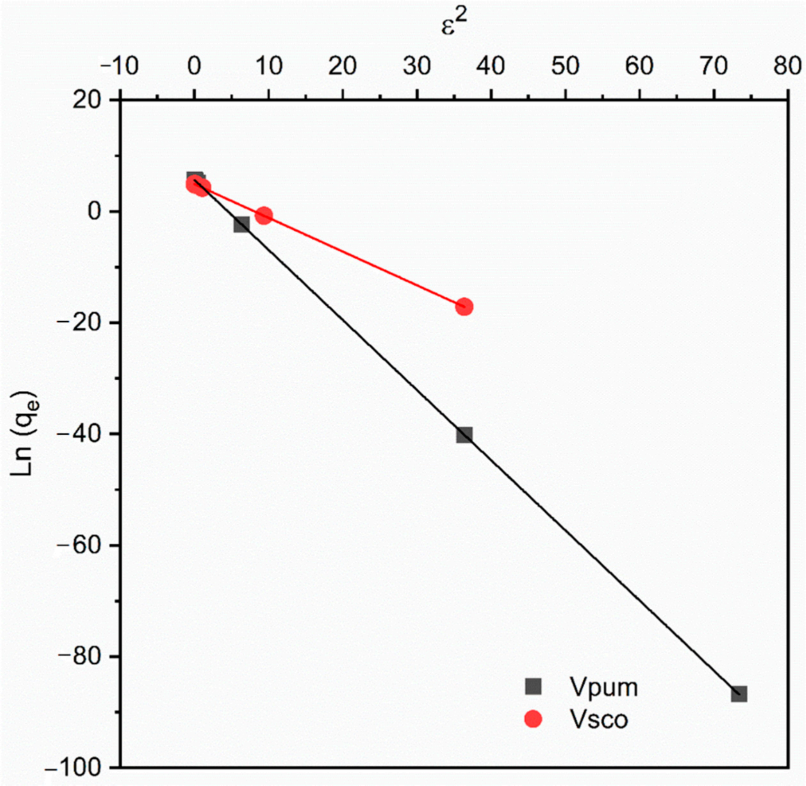

Figure S3. Linear plot of Dubinin-Radushkevich isotherm model for Vpum and Vsco.

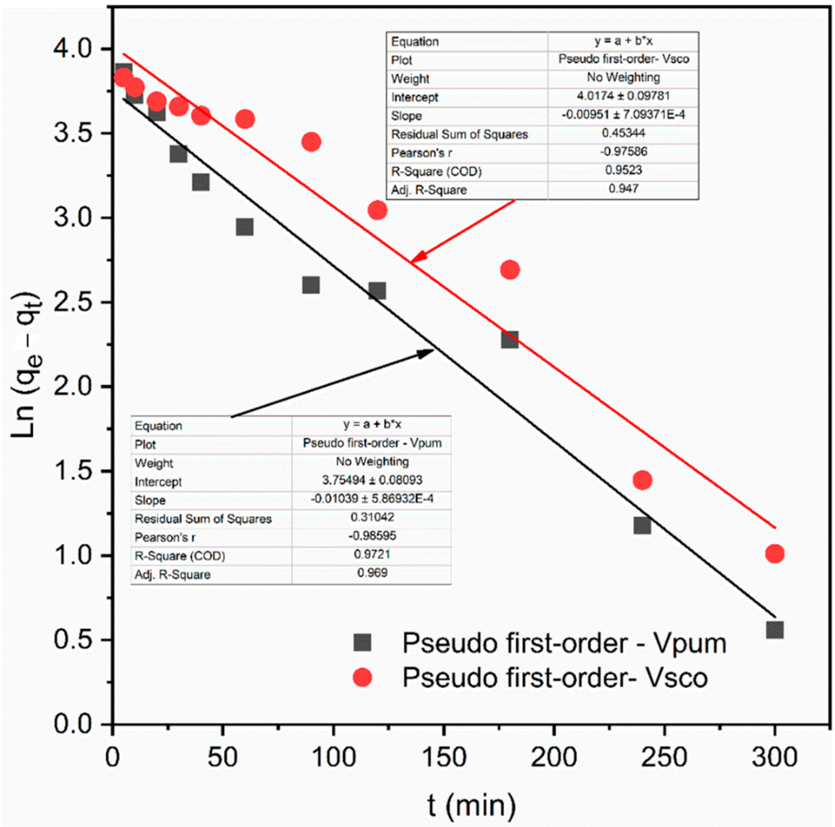

Figure S4. Pseudo first-order kinetic plot for Vpum and Vsco.

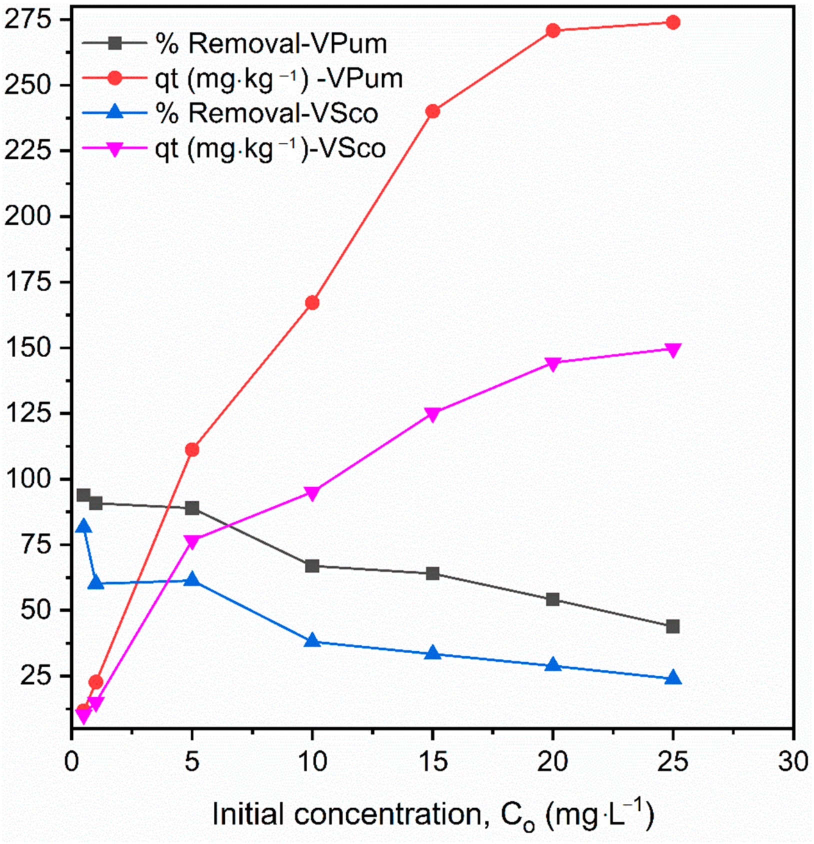

Figure S5. Effects of initial concentration.
